# Polystyrene microplastics reduce the food chain transfer and toxicity of TiO_2_ nanoparticles in crustacean *Artemia salina* through dietary exposure of microalga *Chlorella* sp.: impact of visible light and ultraviolet-A radiation

**DOI:** 10.1039/d6ra05133b

**Published:** 2026-08-18

**Authors:** Camil Rex M, Manshi Kumari Gupta, Chinnappan Sudandiradoss, Amitava Mukherjee

**Affiliations:** a Centre for Nanobiotechnology, VIT Vellore Tamil Nadu India amitav@vit.ac.in amit.mookerjea@gmail.com; b Department of Biotechnology, School of Bio Sciences and Technology, VIT Vellore Tamil Nadu India

## Abstract

Microplastics and nanoparticles are emerging contaminants in the marine system. Although UV-A radiation is environmentally relevant, most ecotoxicity studies overlook its role in modulating contaminant toxicity. Therefore, this study aimed to investigate the trophic transfer potential of titanium dioxide nanoparticles (TiO_2_-NPs) in the absence and presence of amine (NH_2_ MPs) and carboxyl (COOH MPs) functionalized polystyrene microplastics (PS-MPs) under both visible-light and UV-A radiation. The experiments were performed using marine microalgae *Chlorella* sp. and marine crustacean *Artemia salina*, representing the producer-consumer food chain. Bio-uptake of Ti in *Chlorella* sp. was higher under UV-A treatment than under visible-light illumination. Bio-uptake of Ti in *Chlorella* sp. was higher in the absence of PS-MPs. A comparable pattern was observed in the bio-uptake of Ti in *A. salina* following dietary exposure. Consequently, *A. salina* fed with microalgae pre-treated with TiO_2_-NPs reduces survival, enhances oxidative stress, and alters neurotransmitter activity. In contrast, in the presence of PS-MPs, these detrimental effects exhibited by TiO_2_-NPs were reduced. *In silico* studies with acetylcholinesterase provided complementary mechanistic insights into the observed neurotoxicity. Under both light treatments, the biomagnification factor for TiO_2_-NPs and TiO_2_-NPs + PS-MPs was < 1, indicating no biomagnification from *Chlorella* sp. to *A. salina*. Risk quotient analysis revealed the higher ecological risk posed by the TiO_2_-NPs. Pearson correlation and the cluster heatmap revealed that oxidative stress and altered neurotransmitter activity may have impaired *A. salina* survival. Overall, this study shows how light and microplastics modulate the trophic transfer potential and toxicity of TiO_2_-NPs in marine organisms.

## Abstract 2

Illustration of dietary uptake of Ti in *Artemia salina* under visible light and UV-A light.

## Graphical abstract

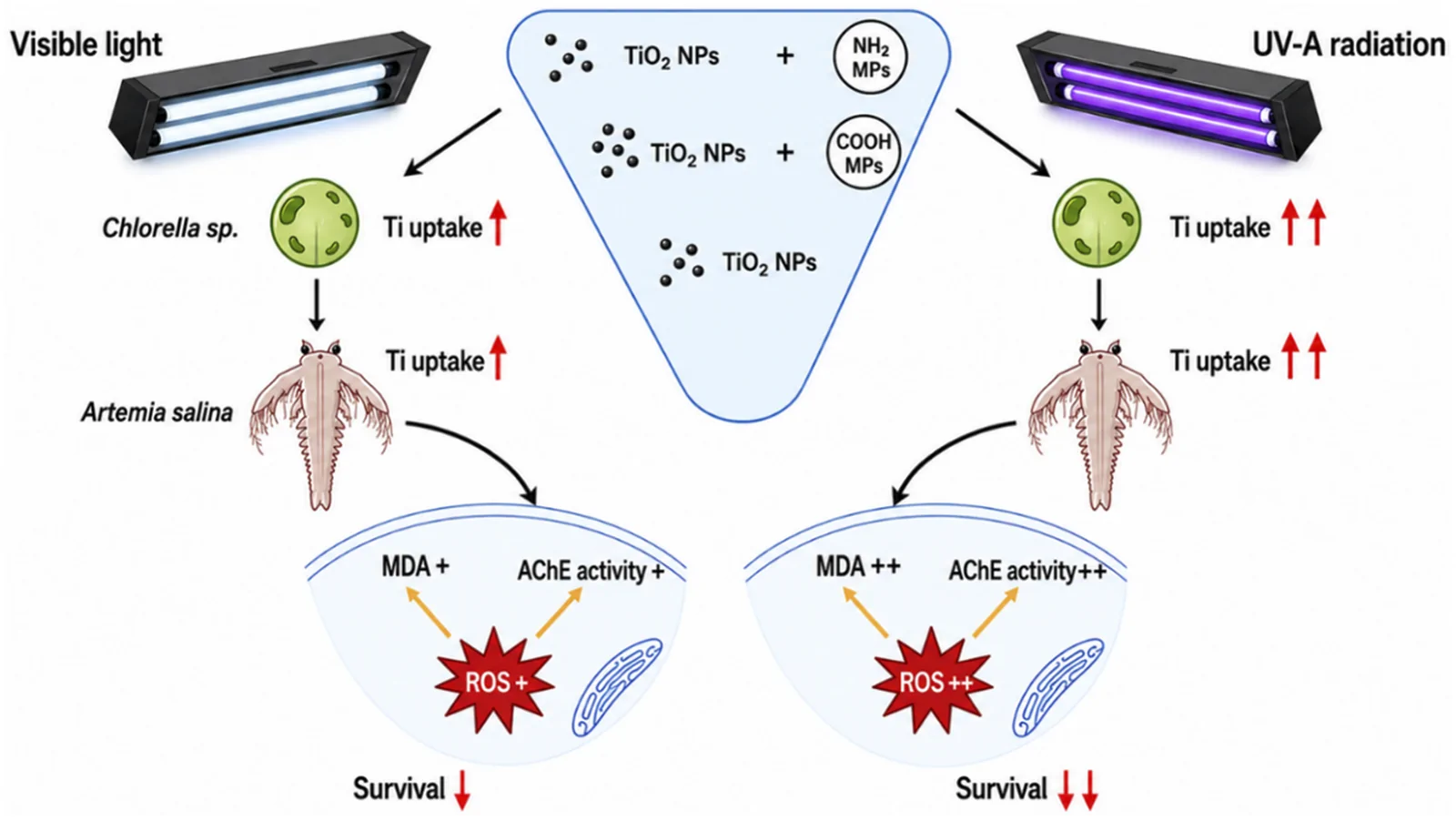

## 1. Introduction

The extensive usage of plastic products, along with their improper waste management, has significantly contributed to marine plastic pollution.^1^ Rivers constitute the primary pathway through which plastic waste enters the ocean, and reports indicate that around 5.25 trillion plastic particles are present in global oceans.^2,3^ These polymers undergo physical, chemical, and biological degradation in the marine system and lead to the formation of smaller plastics (<5 mm) known as microplastics (MPs).^4^ Among the various polymer types, polystyrene is one of the most prevalent and extensively used in disposable food packaging, cosmetics, medical devices, and construction materials.^5^ Amine-functionalized (NH_2_ MPs) and carboxyl-functionalized microplastics (COOH MPs) are among the most commonly used surface-functionalized MPs and are widely utilized in biosensing, drug delivery, and scientific research.^6^ In the marine environment, MPs possess the ability to interact and alter the fate and toxicity of their co-contaminants.^7^ These interactions can substantially alter the bioavailability and ecotoxicological impact of their co-contaminants, such as nanoparticles, towards marine organisms.^8^

Engineered nanoparticles exhibit a steady rise in worldwide production due to their extensive usage in a wide range of applications.^9^ Globally, titanium dioxide nanoparticles (TiO_2_-NPs) are among the most widely produced engineered nanoparticles, with annual production projected to reach 2.5 × 10^6^ metric tons by 2050.^10,11^ TiO_2_-NPs possess exceptional photo-reactivity along with various other distinctive physicochemical properties.^12^ These remarkable properties enable TiO_2_-NPs to be employed in a wide range of applications, including sunscreens, paints, food processing, household products, textiles, photocatalytic technologies, and biomedical research.^11,13^ However, the unregulated release of TiO_2_-NPs into the marine system, along with environmental factors such as ultraviolet-A (UV-A) radiation, can trigger their photocatalytic activity.^14^ Consequently, excessive reactive oxygen species (ROS) are produced, which ultimately induce a range of toxic effects in marine organisms.^15^

As MPs and TiO_2_-NPs co-exist in aquatic systems, there is a significant risk of their transfer across trophic levels from lower to higher organisms.^16,17^ Marine microalgae, as primary producers, form the foundation of the marine food chain and are highly sensitive to environmental contaminants.^18^ Contaminants accumulated by microalgae can subsequently be transferred to primary consumers through dietary exposure.^19^ Therefore, in this study, the marine microalga *Chlorella* sp. was selected as the primary producer (prey), and the microcrustacean *A. salina* was chosen as the primary consumer (predator) to assess the trophic transfer and associated toxicity.

Light plays a crucial role in ecotoxicity assessment, especially when determining the toxicity of photocatalytic materials.^20^ Most scientific investigations utilized visible-light as the light source for trophic transfer studies, often overlooking the impact of environmentally relevant light treatments like UV-A radiation. A few studies have evaluated the effect of UV radiation in altering the waterborne toxicity of nanoparticles and MPs in aquatic organisms.^20–23^ No prior research has evaluated the role of UV radiation in influencing the trophic transfer of contaminants in aquatic biota. Notably, a previous study investigated the trophic transfer of P25 TiO_2_-NPs from marine microalgae *Dunaliella salina* to marine microcrustacean *A. salina* under visible-light treatments.^24^ Similarly, another study evaluated the trophic transfer potential of CuO nanoparticles from freshwater microalgae *Chlorella vulgaris* to freshwater crustacean *Daphnia magna* under visible-light treatments.^25^ However, investigations addressing the trophic transfer of potential nanoparticles in combination with MPs remain scarce. A prior work evaluated the dietary transfer of ZnO nanoparticles together with polystyrene MPs from freshwater microalgae *Chlorella vulgaris* to freshwater crustacean *Daphnia magna* under visible-light exposure.^26^

To the best of our knowledge, no previous work has studied the comparative effects of UV-A illumination and visible-light exposure on the food chain transfer potential of nanoparticles. Notably, evidence concerning the effects of UV-A radiation and visible-light on the food chain transfer potential of nanoparticles in combination with MPs remains scarce. Furthermore, most of the trophic transfer studies evaluate the bioaccumulation and biomagnification potential, often overlooking the underlying mechanism(s) of toxicity assessed through biochemical parameters. Therefore, this investigation aimed to evaluate the effect of UV-A radiation and visible-light on the trophic transfer potential of TiO_2_-NPs alone and their binary mixture with surface functionalized polystyrene MPs (PS-MPs). Our previous study revealed that, due to the comparable size of microalgae *Chlorella* sp. and the PS-MPs, PS-MPs cannot be internalized into the algal cells.^23^ However, PS-MPs can reduce the bioaccumulation of TiO_2_-NPs under both visible and UV-A light, which was also evident from our previous study.^22^ The present study hypothesizes that the elevated photocatalytic potential of TiO_2_-NPs upon UV-A radiation may induce enhanced cellular damage, thereby promoting their increased uptake in microalgal cells relative to visible-light treatment. Furthermore, this uptake of TiO_2_-NPs can be modulated in the presence of functionalized PS-MPs. Consequently, the trophic transfer potential of TiO_2_-NPs towards microcrustaceans is expected to be modulated under UV-A radiation compared to visible-light exposure. The specific objectives of this work were (i) to determine the uptake of TiO_2_-NPs alone and in presence of both NH_2_ MPs and COOH MPs in microalgae *Chlorella* sp. under both visible-light and UV-A radiation; (ii) to assess the trophic transfer potential of TiO_2_-NPs alone and in presence of both NH_2_ MPs and COOH MPs from microalgae to microcrustacean *A. salina* under both visible-light and UV-A radiation; and (iii) to elucidate the mechanism(s) of toxicity in *A. salina* following dietary exposed to microalgae through various biological endpoints. The endpoints include estimation of survival, ROS, malondialdehyde (MDA) levels, neurotransmitter activity, and biomagnification factor (BMF). *In silico* molecular docking and molecular dynamics (MD) simulation were performed for the neurotransmitter acetylcholinesterase (AChE) to understand the molecular basis for the observed neurotoxicity. Risk quotient (RQ) analysis was performed to determine the ecological risk posed by the TiO_2_-NPs. Pearson correlation and cluster heatmap analysis were performed to determine the association among the biological endpoints.

## 2. Materials and methods

### 2.1 Chemicals and characterization of test materials

Commercial TiO_2_ nanoparticles (average diameter: 21 nm) supplied by Sigma-Aldrich (USA) were used. The NH_2_ and COOH PS-MPs (1 µm) were procured from Corpuscular, Inc., USA. In this study, all experiments were performed in artificial seawater (ASW), and its composition is detailed in the SI (SM), Table S1. All other chemical utilized in this study is reported in the SM (Method, S1). The stock solution for TiO_2_-NPs (100 mg L^−1^) was prepared using ultrapure water and subjected to probe sonication for 30 min (Sonic, USA) to obtain a homogeneous dispersion.^22^ Similarly, a 100 mg L^−1^ stock solution was prepared for both NH_2_ and COOH PS-MPs, which were prepared using ultrapure water and subjected to bath sonication (Crest ultrasonics) for 15 min.^22^

The shape and size of the TiO_2_-NPs were examined using a field emission scanning electron microscope (FE-SEM). For the analysis, TiO_2_-NPs powder was placed on the aluminium foil-coated glass slide, and these samples were sputter-coated before imaging. The morphological characteristics and particle size of PS-MPs were determined using FE-SEM in our previous study.^22^ The surface charge and hydrodynamic diameter of TiO_2_-NPs and PS-MPs in the ASW were analysed using a particle size and zeta potential analyser (Brookhaven Instruments, USA).

### 2.2 Test organisms

The present study utilized marine microalgae *Chlorella* sp. and microcrustacean *A. salina* as model organisms. The *Chlorella* sp. was procured from the Central Marine Fisheries Research Institute (CMFRI), Tamil Nadu, India. The microalgae cultures were maintained in Conway medium under fluorescent light with a 16 h light and 8 h dark cycle. To facilitate the hatching of cysts of *A. salina*, filtered and autoclaved natural seawater (NSW) was utilized with a pH of 8.1 ± 0.05. To facilitate hatching, 1 g of dry cysts was introduced into 1 L filtered and autoclaved NSW, with continuous aeration and illumination using fluorescent light. After 24 h, the hatched nauplii were transferred to fresh NSW medium. For the dietary exposure experiments, the 48 h nauplii were used.

### 2.3 Experimental design

The trophic transfer experiments were carried out under two distinct continuous lighting (48 h) conditions at 24 ± 2 °C: (i) visible-light (Philips fluorescent lamp – 400–700 nm; 0.4 mW cm^−2^; exposure dose: 69.12 J cm^−2^; lamp distance 28 cm) and (ii) UV-A exposure (Philips UVA lamp – 320–400 nm; 0.2 mW cm^−2^; exposure dose: 34.56 J cm^−2^; lamp distance 28 cm). Initially, the microalgae *Chlorella* sp. (0.1 optical density) was exposed to both individual and binary mixtures of contaminants under (i) visible-light, and (ii) UV-A treatments. For individual contaminant exposure, the microalgae *Chlorella* sp. was exposed to two concentrations (2.5 and 10 mg L^−1^) of PS-MPs (NH_2_ MPs and COOH MPs), and one concentration of TiO_2_-NPs (1 mg L^−1^). Although internalization of PS-MPs is not possible in *Chlorella* sp. due to their comparable size, it was still exposed to *Chlorella* sp. to evaluate whether any biochemical alterations could occur in *A. salina* following dietary exposure. Furthermore, for the binary mixture exposure, each concentration of NH_2_ MPs and COOH MPs was separately combined with 1 mg L^−1^ TiO_2_-NPs. The projected environmental concentrations of TiO_2_-NPs have been reported up to mg L^−1^.^27^ Reports indicate that MPs levels in marine environments may reach concentrations of up to 0.1 to 100 mg L^−1^.^28^ In addition, environmentally realistic scenarios such as accidental spills may lead to a drastic increase in TiO_2_-NPs and MPs concentrations in the aquatic system.^29,30^ Therefore, the exposure concentrations selected in the present study were intended to represent worst-case yet environmentally plausible exposure scenarios. Following the exposure period (48 h), the microalgal cells that were exposed to contaminants under visible-light treatments were washed several times with fresh ASW by centrifugation (7000 rpm, 4 °C). Subsequently, only the washed cell pellets were fed to *A. salina* under visible-light illumination for 48 h to evaluate the trophic transfer potential of TiO_2_-NPs both in the absence and presence of PS-MPs. Similarly, microalgal cells exposed that were exposed to contaminants under UV-A were washed several times with fresh ASW by centrifugation (7000 rpm, 4 °C). Subsequently, only the washed cell pellets were fed to *A. salina* under UV-A conditions for 48 h to assess the trophic transfer potential of TiO_2_-NPs and their combination with PS-MPs.

#### 2.3.1 Uptake of Ti in *Chlorella* sp

Ti uptake in *Chlorella* sp. exposed to TiO_2_-NPs alone and co-exposure with PS-MPs was quantified after 48 h of exposure under visible-light and UV-A irradiation. Following the exposure period, the experimental samples were subjected to centrifugation (7000 rpm; 10 min), and the pellets were washed with EDTA (0.02 M) to eliminate the surface-bound TiO_2_-NPs. After washing, the experimental samples were subjected to acid digestion using conc.HNO_3_, and the digested samples were analyzed to determine the Ti concentration using atomic absorption spectroscopy (AAS).

#### 2.3.2 Dietary uptake, accumulation, and determination of the biomagnification factor

To determine Ti uptake in *A. salina* following dietary exposure, the test groups were fed with microalgae pre-treated with TiO_2_-NPs alone and TiO_2_-NPs + PS-MPs under both visible-light and UV-A treatment. After a 48 h period, the samples were washed and subjected to acid digestion with conc.HNO_3_, and the digested samples were analyzed using AAS to determine the Ti concentration. For the accumulation experiments, following the dietary exposure period, *A. salina* was transferred to a fresh ASW medium for 24 h to facilitate the depuration of TiO_2_-NPs. Following depuration, the nauplii were collected and subjected to acid digestion with conc.HNO_3_ and subjected to AAS analysis for quantification of Ti concentrations. To examine the dietary transfer of TiO_2_-NPs in the absence and presence of PS-MPs from microalgae to brine shrimp were determined by computing BMF using the following formula,^31^ BMF = concentration of Ti in consumer/concentration of Ti in producer.

#### 2.3.3 Survival estimation of *Artemia salina* following dietary exposure

The survival of *A. salina* nauplii (*n* = 10) fed with microalgae pre-treated with TiO_2_-NPs alone, PS-MPs alone, and TiO_2_-NPs + PS-MPs was evaluated after a 48 h exposure period. Following the exposure period, the nauplii were subjected to microscopic analysis, and the nauplii exhibiting movements were considered surviving/alive organisms.^32^

#### 2.3.4 Oxidative stress estimation in *Artemia salina* following dietary exposure

The total ROS levels in *A. salina* (*n* = 200), followed by dietary exposure to contaminated microalgae, were assessed using non-fluorescent dye 2′,7′-dichlorodihydrofluorescein diacetate. After the dietary exposure period (48 h), the *A. salina* nauplii were homogenized, centrifuged (7000 rpm; 10 min; 4 °C), and the subsequent supernatant was used for ROS measurements.^33^ After the dietary exposure period, the MDA levels in *A. salina* (*n* = 200) were evaluated using the TBA-TCA method.^32^ The detailed protocol for ROS and MDA measurements is incorporated in the SM (Method S2).

#### 2.3.5 *In vitro* AChE activity and *in silico* molecular docking and molecular dynamics simulation of TiO_2_ with AChE

The AChE activity of *A. salina* nauplii (*n* = 200) fed with microalgae pre-treated with TiO_2_-NPs alone, PS-MPs alone, and TiO_2_-NPs + PS-MPs was evaluated after a 48 h exposure period. Subsequent to the exposure period, homogenization of the experimental samples were performed, and the supernatant was utilized to evaluate the AChE activity following the procedure detailed in SM (Method, S3).^34^

For the *in silico* analysis, structural models were obtained from publicly available databases.^35–37^ Since no experimentally resolved crystal structure of *Artemia salina* AChE is currently available in the Protein Data Bank, the crystal structure of AChE (PDB ID: 7RB7; resolution: 2.60 Å) was selected as a representative model for investigating the potential interaction of TiO_2_ with AChE at the molecular level.^12^ The inorganic ligand, titanium dioxide (PubChem CID: 26042), was obtained in a two-dimensional (2D) format and converted into a three-dimensional (3D) structure using Open Babel.^10,11^ Owing to the limitations of conventional molecular docking in representing an entire nanoparticle surface, TiO_2_ was modelled as a simplified inorganic ligand to provide qualitative insights into its potential interactions with the AChE binding site. The detailed molecular docking and molecular dynamics simulation procedures are provided in the SM (Method, S4).

### 2.4 Environmental risk quotient

Risk quotient analysis is an approach for determining the risk posed by contaminants in the aquatic system. In this study, the ecological risk posed by the TiO_2_-NPs was estimated using risk quotient analysis. To determine the risk quotient of TiO_2_-NPs, the following formula was used,

1 Predicted no-effect concentration = EC_50_/assessment factor

2 RQ = Measured environmental concentration/predicted no-effect concentration

Additional details of the risk quotient analysis were provided in the SM (Method, S5).

### 2.5 Pearson correlation, cluster heatmap, and data analysis

To evaluate the association between various biological endpoints, the Pearson correlation and cluster heatmap analyses were performed using OriginPro. The statistical analysis was performed using GraphPad Prism 8.0, and the statistical difference between the pristine and the mixture group was analyzed using a two-way ANOVA with a Bonferroni post-test. All tests were conducted in triplicate (*n* = 3), and the findings were reported as mean ± standard deviation.

## 3. Results

### 3.1 Characterization

The FE-SEM analysis (SM, Fig. S1) revealed that TiO_2_-NPs were 21 nm and spherical in morphology. Similarly, our previous study revealed that the PS-MPs were spherical in morphology and 1.9 µm.^22^ Fig. S2A shows the hydrodynamic size of TiO_2_-NPs, PS-MPs, and their co-exposure groups in ASW. PS-MPs exhibited a larger hydrodynamic size than pristine TiO_2_-NPs. Moreover, PS-MPs (10 mg L^−1^) exhibited a larger hydrodynamic size than PS-MPs (2.5 mg L^−1^). In contrast to TiO_2_-NPs, TiO_2_-NPs co-exposed with NH_2_ MPs showed a substantial increase in hydrodynamic size. A comparable pattern was observed for the TiO_2_-NPs co-exposed with COOH MPs. The initial and final zeta potential of TiO_2_-NPs, PS-MPs, and their co-exposure groups in ASW under both visible-light and UV-A exposure are provided in Fig. S2B-D. The initial zeta potential results revealed that TiO_2_-NPs and NH_2_ MPs exhibited a positive surface charge. In contrast, COOH MPs showed a negative surface charge. The co-exposure of TiO_2_-NPs with NH_2_ MPs showed less positive charge compared to TiO_2_-NPs. Similarly, TiO_2_-NPs co-exposed with COOH MPs exhibited more decrease in negative charge compared to COOH MPs. A similar trend was observed under both light conditions after 48 h, with much more reduction under UV-A treatment than under visible-light conditions.

### 3.2 Bio-uptake of Ti in microalgae *Chlorella* sp.

Fig. 1A and B show the uptake of Ti in *Chlorella* sp. following treatment with TiO_2_-NPs alone and TiO_2_-NPs + PS-MPs under visible-light and UV-A treatment, respectively. Exposure of TiO_2_-NPs alone exhibited a significant Ti uptake (*p* < 0.001) relative to TiO_2_-NPs + PS-MPs in *Chlorella* sp. under both visible and UV-A treatment. Under UV-A treatment, the Ti uptake in microalgae was higher with respect to visible-light following treatment with TiO_2_-NPs alone. Notably, Ti uptake upon exposure to TiO_2_-NPs + PS-MPs was higher under UV-A treatment with respect to visible-light treatment. Furthermore, the binary mixture of TiO_2_-NPs + 2.5 mg L^−1^ PS-MPs showed a significant increase in Ti uptake than the binary mixture of TiO_2_-NPs + 10 mg L^−1^ PS-MPs under both light treatments (*p* < 0.001). The joint exposure to TiO_2_-NPs + NH_2_ MPs showed more Ti uptake in the microalgal cells relative to the joint exposure to TiO_2_-NPs + COOH MPs, irrespective of the light treatments.

**Fig. 1:**
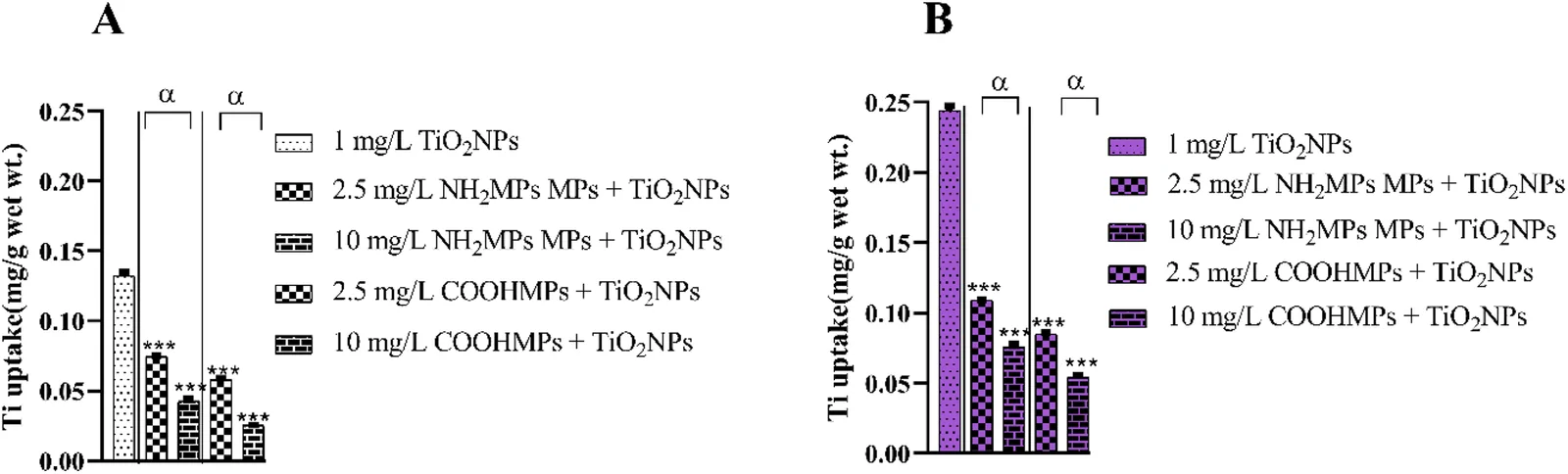
The uptake of Ti by *Chlorella* sp. exposed to TiO_2_-NPs alone and combined with functionalized PS-MPs under (A) visible-light and (B) UV-A treatments. Statistical differences between the pristine TiO_2_-NPs and their binary exposure groups are represented in asterisks (***p < 0.001). The statistical differences among the co-exposure sets are indicated by α (*p* < 0.001).

### 3.3 Bio-uptake and accumulation of Ti in *Artemia salina*

Fig. 2A and B show the Ti uptake and accumulation in *A. Salina* following dietary exposure to microalgae pre-treated with TiO_2_-NPs alone, TiO_2_-NPs + PS-MPs under visible-light and UV-A exposure, respectively. The dietary exposure of pristine TiO_2_-NPs pre-treated microalgae exhibited more Ti uptake in *A. salina* under UV-A exposure than under visible-light treatments. The microalgae pre-treated with the combination of TiO_2_-NPs and PS-MPs showed significantly (*p* < 0.001) reduced Ti uptake in *A. salina* than those exposed to TiO_2_-NPs alone under both light treatments. Furthermore, dietary exposure of microalgae treated with TiO_2_-NPs in the presence of PS-MPs showed more Ti uptake in *A. Salina* under UV-A exposure than in visible-light treatments. The *A. salina* fed with microalgae pre-treated with TiO_2_-NPs + NH_2_ MPs showed increased Ti uptake than those fed with microalgae pre-treated with TiO_2_-NPs + COOH MPs. The dietary exposed organisms exhibited the following trend in the Ti uptake under both UV-A and visible-light exposure: TiO_2_-NPs > TiO_2_-NPs with PS-MPs 2.5 mg L^−1^ > TiO_2_-NPs with 10 mg L^−1^ PS-MPs. Regardless of light condition, the accumulation results exhibited a similar trend of Ti uptake, with a significant reduction (*p* < 0.001) in Ti concentrations compared to uptake.

**Fig. 2:**
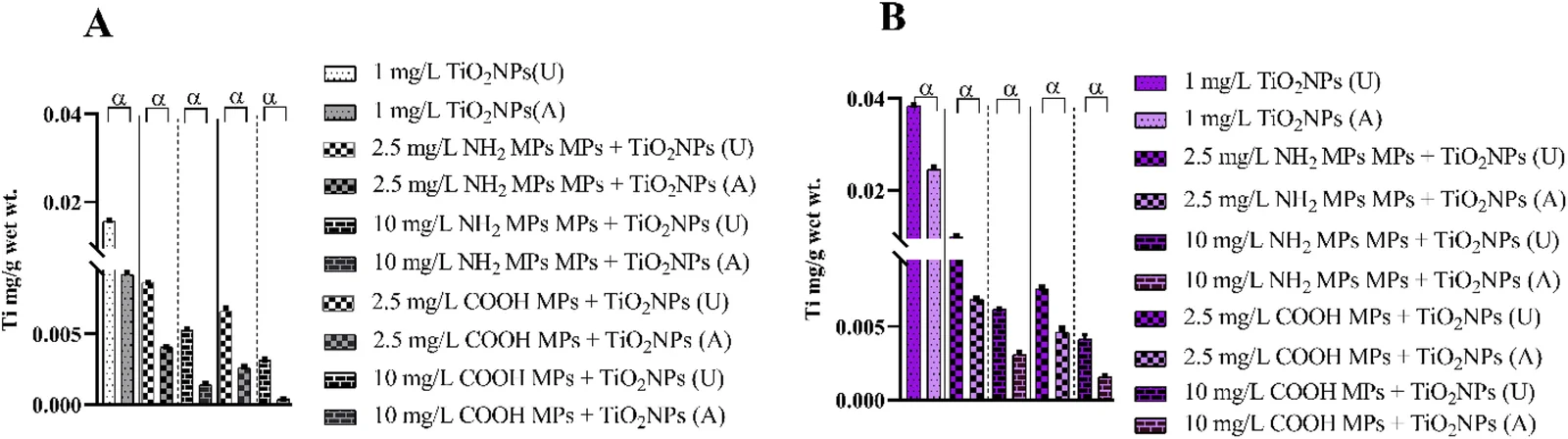
The uptake (U) and accumulation (A) of Ti in *A. salina* nauplii upon dietary exposure to TiO_2_-NPs alone and their joint exposure with PS-MPs, under (A) visible-light; (B) UV-A irradiation. The statistical differences between uptake and accumulation among test sets are represented by α (*p* < 0.001).

### 3.4 Effects on the survival of *Artemia salina*

Fig. 3A and B show the survival of *A. salina* fed with *Chlorella* sp. pre-treated with TiO_2_-NPs alone, PS-MPs alone, and their co-exposure under visible-light and UV-A light treatments, respectively. Under both visible and UV-A light treatments, dietary exposure of microalgae pre-treated with TiO_2_-NPs exhibited a substantial diminution in survival of *A. salina* with respect to the control (*p* < 0.001). Notably, dietary-exposed TiO_2_-NPs showed a more pronounced decline in the survival percentage of *A. salina* under UV-A light than those exposed under visible-light treatments. Nevertheless, no significant decline in the survival of the organisms was observed following dietary exposure to microalgae pre-treated with PS-MPs under both UV-A and visible-light treatments, with respect to the control (*p* > 0.05). The same trend was evident in organisms fed with microalgae pre-treated with TiO_2_-NPs + PS-MPs, under both light treatments.

**Fig. 3:**
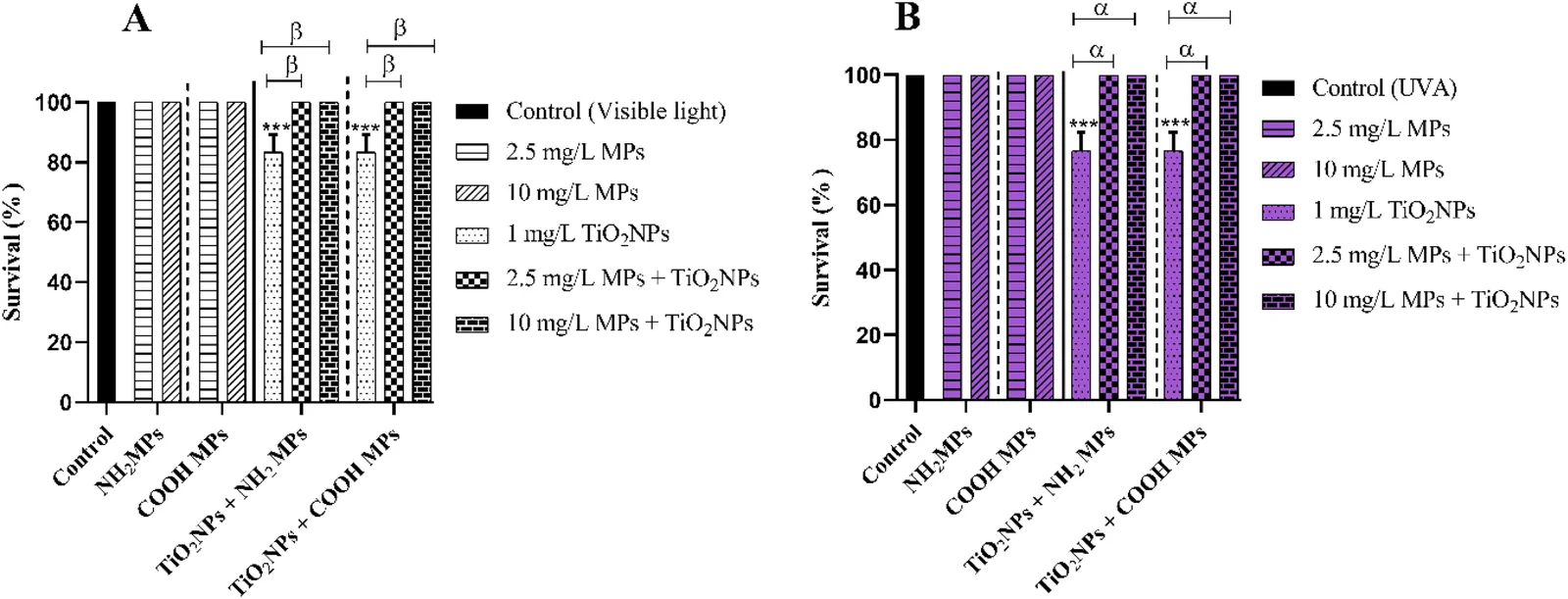
Survival percentage of *A. salina* upon dietary exposure to TiO_2_-NPs alone, functionalized PS-MPs alone, and their co-exposure under (A) visible-light; (B) UV-A irradiation. Statistical changes relative to control and exposure sets are represented as asterisks (***p < 0.001); the statistical differences among the individual and co-exposure sets are indicated by α: (*p* < 0.001) and β: (*p* < 0.01).

### 3.5 Effects on ROS generation in *Artemia salina*

Fig. 4A and B depict the ROS generation in *A. salina* following dietary exposure to *Chlorella* sp. pre-treated with TiO_2_-NPs alone, PS-MPs alone, and their co-exposure under visible-light and UV-A illumination, respectively. In contrast to the control, dietary exposure to TiO_2_-NPs pre-treated microalgae revealed a significant increment in ROS generation in *A. salina* under both light treatments (*p* < 0.001). Furthermore, ROS generation was more pronounced in organisms exposed to TiO_2_-NPs under UV-A radiation than under visible-light treatments. Nevertheless, ROS production did not differ significantly in the test organism fed with microalgae pre-treated with PS-MPs alone, and co-exposed to TiO_2_-NPs with 10 mg L^−1^ COOH MPs (*p* > 0.05). Compared to the test groups fed with TiO_2_-NPs alone-pretreated microalgae, the test groups fed with microalgae pre-treated with TiO_2_-NPs + PS-MPs exhibited a significant decrease in ROS generation. The dietary exposure of binary mixtures pre-treated microalgae to *A. salina* under UV-A treatments showed elevated ROS production than those under visible-light treatments.

**Fig. 4:**
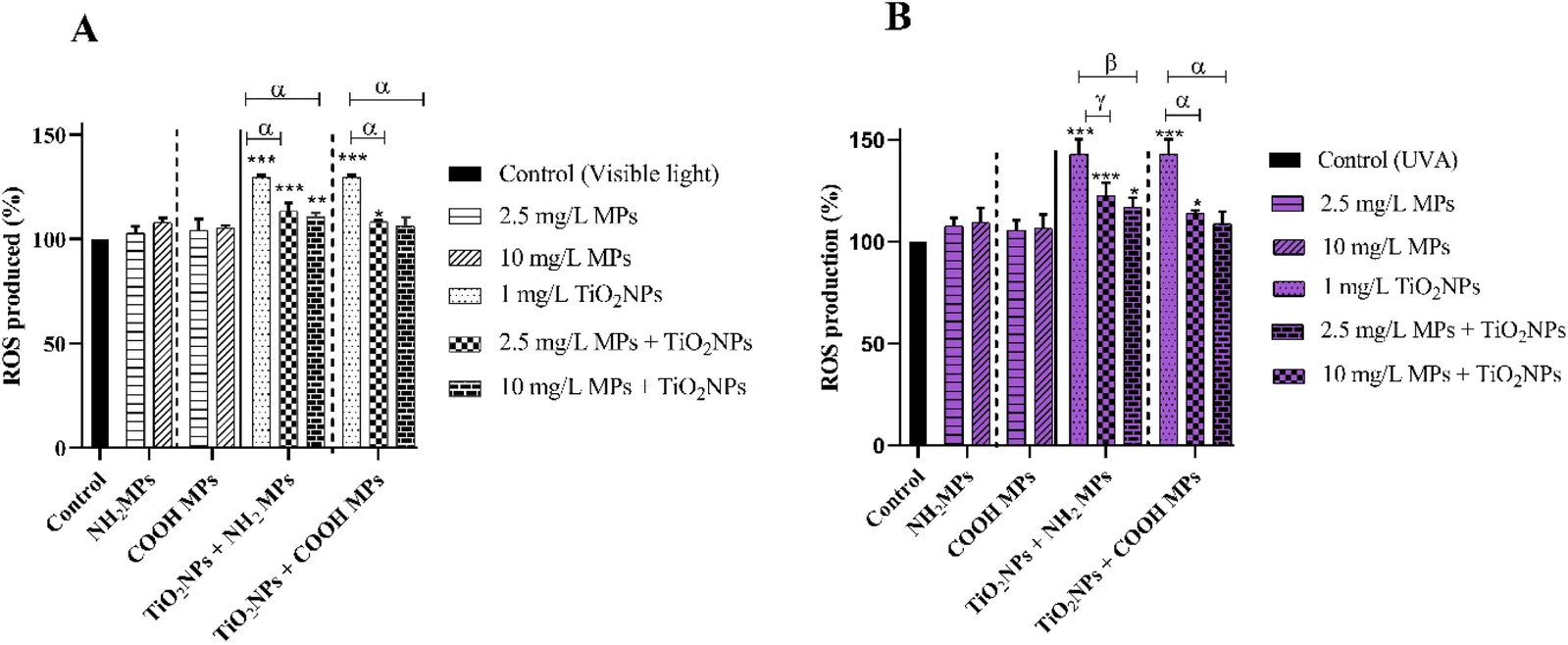
Changes in the ROS production in *A. salina* upon dietary exposure to individual TiO_2_-NPs alone, functionalized PS-MPs alone, and their co-exposure under (A) visible-light; (B) UV-A irradiation. Statistical changes relative to control and exposure sets are represented by asterisks (***p < 0.001 and * < 0.05); the statistical differences among the individual and co-exposure sets are represented as α: (*p* < 0.001), β: (*p* < 0.01), and γ: (*p* < 0.05).

### 3.6 Effects on the MDA levels in *Artemia salina*

Fig. 5A and B show the MDA content in *A. salina* following dietary exposure to *Chlorella* sp. pre-treated with TiO_2_-NPs alone, PS-MPs alone, and their co-exposure under visible-light and UV-A illumination, respectively. Regardless of the light treatments, all test groups showed a significant enhancement in the MDA content relative to the control sets, except for *A. salina* fed with microalgae pre-treated with PS-MPs alone and TiO_2_-NPs + 10 mg L^−1^ COOH MPs. The organism fed with microalgae pre-treated with TiO_2_-NPs under UV-A exposure exhibited higher MDA levels than the experimental groups under visible-light. *A. salina* fed with microalgae pre-treated with binary mixtures exhibited a significant reduction in MDA levels compared to *A. salina* fed with TiO_2_-NPs alone pre-treated microalgae (*p* < 0.001). The *A. salina* fed with microalgae pre-treated with TiO_2_-NPs + PS-MPs under UV-A treatments showed an increased MDA content in *A. salina* relative to the groups maintained under visible-light illumination.

**Fig. 5:**
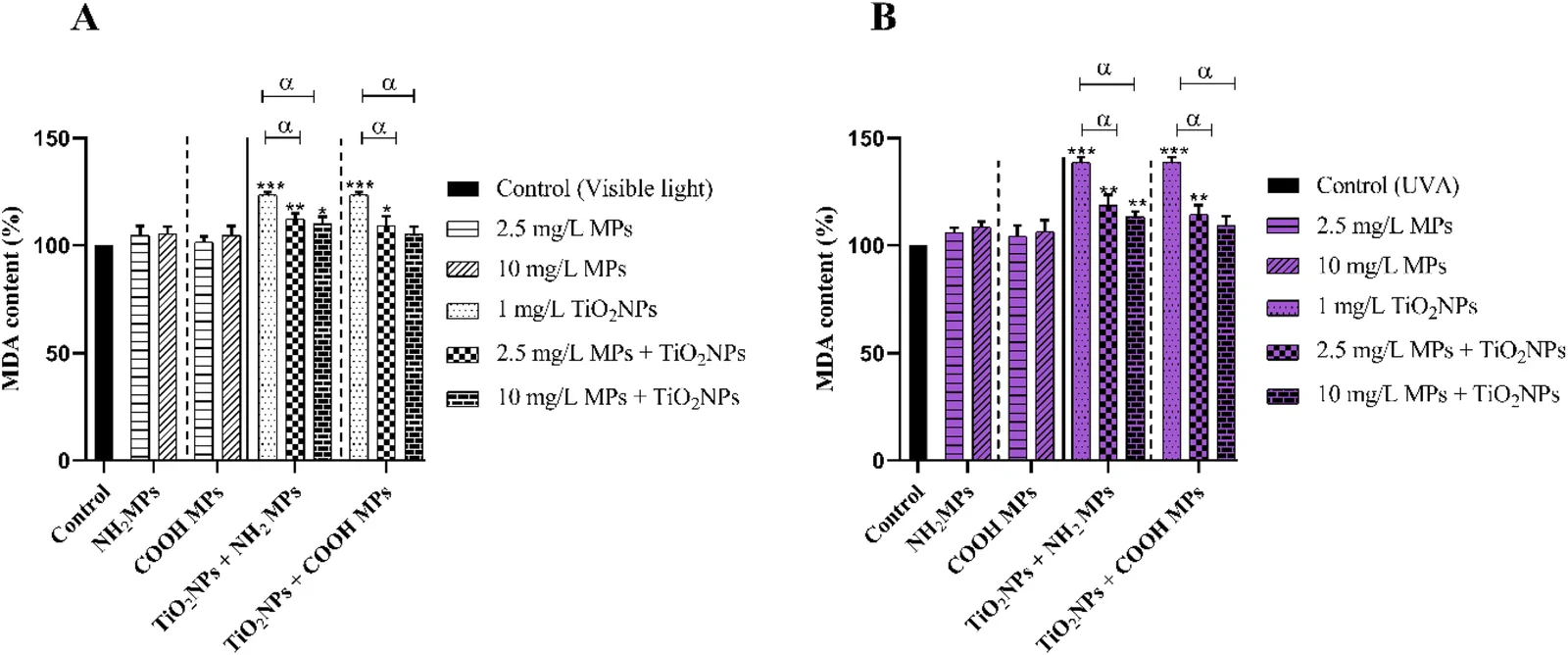
MDA level of *A. salina* upon dietary exposure to TiO_2_-NPs alone, functionalized PS-MPs alone, and their co-exposure under (A) visible-light; (B) UV-A irradiation. Statistical changes relative to control and exposure sets are represented by asterisks (***p < 0.001, **p < 0.01, and * *p* < 0.05); the statistical differences among the individual and co-exposure sets are represented as α (<0.001).

### 3.7 Effects on the AChE activity in *Artemia salina*

#### 3.7.1 In vitro

Fig. 6A and B depict the AChE activity of *A. salina* fed *Chlorella* sp. that had been pre-treated with TiO_2_-NPs alone, PS-MPs alone, and their joint exposure under visible-light and UV-A light treatments, respectively. In contrast to the control, dietary exposure to TiO_2_-NPs alone revealed a significant increment in AChE activity under visible as well as UV-A light treatments (*p* < 0.001). Notably, the observed effects were more prominent under UV-A irradiation. In both light treatments, no significant alterations in AChE activity were observed in *A. salina* fed with microalgae pre-treated with PS-MPs alone and TiO_2_-NPs + PS-MPs, in contrast to control sets (*p* > 0.05).

**Fig. 6:**
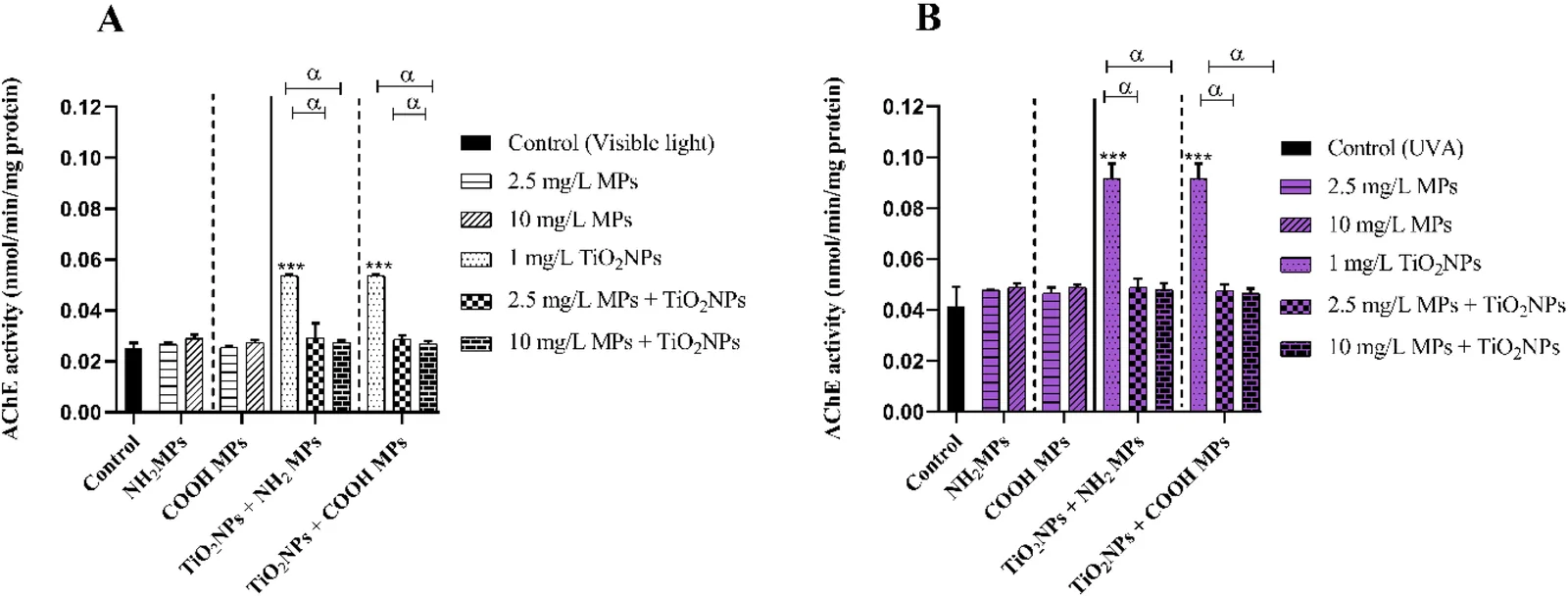
The AChE enzyme activity of *A. salina* upon dietary exposure to TiO_2_-NPs alone, functionalized PS-MPs alone, and their co-exposure under (A) visible-light; (B) UV-A irradiation. Statistical changes relative to control and exposure sets are represented by asterisks (***p < 0.001); the statistical differences among the individual and co-exposure groups are represented as α: (*p* < 0.001).

#### 3.7.2 In Silico

To investigate the interaction potential of TiO_2_ with AChE, the active site architecture of the enzyme was initially characterized. The analysis revealed a large and well-defined binding pocket comprising 27 amino acid residues, including ALA117, GLY118, SER121, GLY122, ALA123, and ALA130. Notably, this pocket exhibited a high binding probability (0.964) along with a moderate conservation score (0.729), indicating that it is both structurally accessible and functionally relevant. Taken together, these features suggest that AChE may provide a favorable microenvironment for potential interactions with TiO_2_. Building on this structural insight, molecular docking was performed to evaluate the binding of TiO_2_ within the predicted active site. The AChE-TiO_2_ complex demonstrated a binding affinity of −3.43 kcal mol^−1^ (Fig. 7A), suggesting a potential interaction. A closer examination of the binding interface revealed that key residues, including ALA117, GLY118, SER121, GLY122, PHE295, and TYR341, were predicted to participate in the interaction with the complex through hydrogen bonding and van der Waals interactions. Importantly, these residues largely overlap with those identified in the active site prediction, thereby suggesting that TiO_2_ may interact within the functional core of the enzyme. This observation suggests that the predicted interaction may influence the structural dynamics of AChE.

**Fig. 7:**
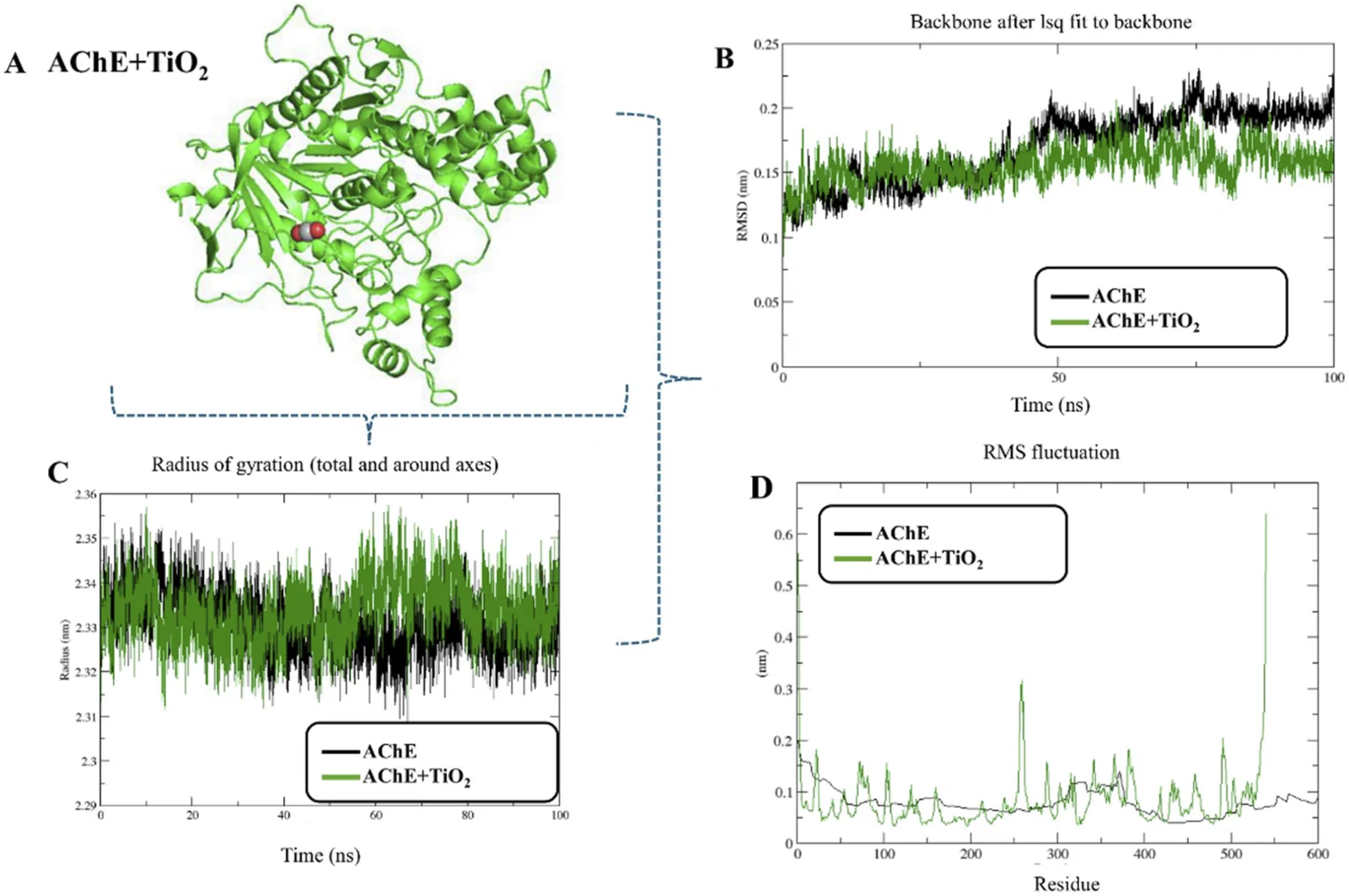
Structural and dynamic analysis of AChE in the absence and presence of TiO_2_ (A) Docked structure of the AChE-TiO_2_ complex showing the TiO_2_ positioned within the predicted active site pocket; (B) backbone RMSD profiles over a 100 ns molecular dynamics simulation for AChE (black) and AChE-TiO_2_ complex (green), indicating reduced structural deviation upon nanoparticle binding; (C) radius of gyration (*R*_g_) as a function of simulation time, demonstrating maintained or slightly enhanced compactness of AChE in the presence of TiO_2_; (D) residue-wise RMSF comparison highlighting decreased atomic fluctuations in the AChE-TiO_2_ complex relative to the wild type, suggesting increased structural stability upon interaction with TiO_2_.

To further probe this possibility, molecular dynamics simulations (100 ns) were performed to evaluate the structural stability of the predicted TiO_2_-AChE complex. Structural deviations and flexibility were quantified using RMSD, RMSF, and radius of gyration (*R*_g_) (Fig. 7B–D). In its native state, AChE displayed an average RMSD of ∼0.20 nm, RMSF of ∼0.18 nm, and *R*_g_ of ∼2.33 nm, reflecting a moderately flexible yet stable structure. The observed fluctuations are primarily associated with loop regions and peripheral domains, which are known to contribute to the intrinsic dynamic behavior required for enzymatic function. In contrast, upon the predicted interaction with TiO_2_, a consistent reduction in structural fluctuations was observed across all parameters. Specifically, the AChE-TiO_2_ complex exhibited a lower RMSD (∼0.15 nm) and RMSF (∼0.10 nm), indicating enhanced structural stability and reduced residue-level mobility (Fig. 7B and D). Concurrently, the *R*_g_ profile remained relatively stable with a slight tendency toward reduced values (∼2.31–2.33 nm), suggesting that the overall compactness of the protein is preserved or marginally improved (Fig. 7C). These coordinated changes collectively suggest a potential stabilization effect associated with the predicted TiO_2_-AChE interaction.

### 3.8 Biomagnification factor of TiO_2_-NPs in the presence and absence of PS-MPs

Table 1 shows the BMF of TiO_2_-NPs in the presence and absence of PS-MPs from *Chlorella* sp. to *A. salina* under both UV-A and visible-light. The highest BMF was observed for the dietary exposure of TiO_2_-NPs under UV-A treatments. However, there was no significant difference (*p* > 0.05) in the BMF of TiO_2_-NPs, both in the presence and absence of PS-MPs.

**Table 1:** Biomagnification factor of TiO_2_-NPs in the presence and absence of PS-MPs from *Chlorella* sp. to *A. salina* under both UV-A and visible-light illumination

Contaminant	UV-A	Visible-light
TiO_2_-NPs 1 mg L^−1^	0.1 ± 0.002	0.069 ± 0.0008
2.5 mg L^−1^ NH_2_ MPs + TiO_2_-NPs	0.062 ± 0.0009	0.054 ± 0.0009
10 mg L^−1^ NH_2_ MPs + TiO_2_-NPs	0.041 ± 0.002	0.033 ± 0.003
2.5 mg L^−1^ COOH MPs + TiO_2_-NPs	0.054 ± 0.002	0.045 ± 0.001
10 mg L^−1^ COOH MPs + TiO_2_-NPs	0.029 ± 0.001	0.014 ± 0.001

## 4. Discussion

TiO_2_-NPs and PS-MPs are widely distributed in marine environments.^38,39^ Although the selected exposure concentrations are higher than the commonly reported concentrations, these concentrations can be plausible exposure scenarios in localized contamination hotspots and during accidental release.^27–30^ The bioaccumulation of contaminants can be influenced by various factors, including light, contaminant size, concentration, co-pollutants, and various other physicochemical properties of the contaminants.^40–42^ TiO_2_-NPs are highly photoreactive under UV-A exposure due to their wide band gap energy, which enables excessive electron–hole pair generation and consequently augments ROS generation.^43^ In the present study, the bio-uptake of Ti was higher in microalgal cells under UV-A illumination relative to visible-light. This increment could be attributed to the excess generation of free radicals and associated cell membrane damage, which may have facilitated higher Ti bio-uptake in microalgal cells under UV-A treatments. A previous study has also reported that under UV-A treatments, TiO_2_-NPs induced substantial cell damage to marine microalgae *Phaeodactylum tricornutum*.^21^ Our previous study revealed that the microalgae used in this study, *Chlorella* sp., cannot internalize PS-MPs because of their comparable size.^23^ However, plastics can influence the bioavailability of their co-contaminants due to their exceptional sorption properties.^44^ The current study revealed that the bio-uptake of Ti was higher in algal cells on exposure to TiO_2_-NPs alone than in combination with PS-MPs. This could be attributed to the reduced bioavailability of TiO_2_-NPs in the presence of PS-MPs, which was also evident from our previous study.^22^ Similarly, a previous investigation revealed that MPs reduced the bioavailability of ZnO nanoparticles towards marine microalgae *Tetraselmis helgolandica*.^44^ Furthermore, this study exhibited a more reduction in the Ti bio-uptake into the algal cells on exposure to COOH MPs + TiO_2_-NPs than NH_2_ MPs + TiO_2_-NPs. This comparable reduction in the bio-uptake can be associated with the hetero-aggregation of the pollutant mixture, attributed to the strong electrostatic attraction between oppositely charged COOH MPs and TiO_2_-NPs.^22^

In the marine system, microalgae are exposed to a wide range of contaminants.^45^ Consequently, these contaminants can be transferred to various higher trophic level organisms through the dietary uptake of contaminated microalgae. The present study revealed the trophic transfer of TiO_2_-NPs from marine microalgae *Chlorella* sp. to marine crustacean *A. salina*. In accordance with our findings, a prior work revealed the trophic transfer potential of TiO_2_-NPs from freshwater microalgae *Chlorella pyrenoidosa* to freshwater crustacean *Daphnia magna*.^46^ As expected, the dietary exposure of pristine TiO_2_-NPs pre-treated algal cells showed higher Ti bio-uptake in *A*. *salina* when compared to the dietary exposure of binary mixture pre-treated algal cells. Furthermore, pristine and binary mixture pre-treated algal cells under UV-A exposure showed higher Ti bio-uptake in *A. salina* than those under visible-light treatments. These trends explicitly revealed that Ti bio-uptake patterns in *Chlorella* sp. are strongly in alignment with dietary bio-uptake of Ti in *Artemia salina*. Consequently, *A. salina* fed with algal cells pre-treated with TiO_2_-NPs alone exhibited a significant reduction in survival under both light treatments, with a more pronounced decrease observed under UV-A exposure. Though the binary mixture pre-treated algal cells showed uptake in *A. salina*, there was no significant alteration in their survival. This indicates that the uptake levels of Ti may not be sufficient to induce mortality but may induce sublethal toxicity.

ROS and MDA are important markers for the oxidative stress, especially for contaminants with photocatalytic activity.^47^ The current study showed enhanced ROS production in *A. salina* followed by their dietary exposure to microalgal cells pretreated with TiO_2_-NPs alone under both light treatments. Notably, the pronounced ROS production in *A. salina* fed with microalgal cells pre-treated with TiO_2_-NPs under UV-A exposure may be associated with more uptake of Ti in algal cells than under visible-light treatments. This excessive ROS production can lead to membrane damage in *A. salina*, which was evident from MDA level estimation. A prior study reported that MDA production was increased in the fish *Clarias gariepinus* fed with TiO_2_-NPs pre-treated with cyclopoid copepod sp.^48^ Interestingly, though the survival of *A. salina* fed with the binary mixture pre-treated microalgae was not affected, oxidative stress production was observed. This indicated the sub-lethal toxicity of dietary-exposed TiO_2_-NPs + PS-MPs. Furthermore, the insignificant oxidative stress exhibited by the dietary-exposed TiO_2_-NPs + 10 mg L^−1^ COOH MPs could be attributed to the low levels of Ti uptake, which may not have been sufficient to induce oxidative stress. Overall, the findings of ROS generation were strongly consistent with those of MDA production.

Elevated oxidative stress may be associated with alterations in neurotransmitter activity in *A. salina*. AChE is a widely used biomarker for neurotoxicity, as it is associated with cholinergic neurotransmission.^49^ It is mainly responsible for behavioral changes, locomotory functions, and can ultimately impact the survival of the organism.^50,51^ In this study, the findings of AChE activity revealed that dietary exposure to TiO_2_-NPs alone affected AChE activity, which also aligned with survival results. Furthermore, *in silico* docking and MD simulations were performed to provide complementary mechanistic insights into the observed neurotoxicity of TiO_2_-NPs in *A. salina*. Molecular docking suggests that TiO_2_ may interact with the predicted active site of AChE, with a binding affinity of −3.43 kcal mol^−1^. This interaction involves critical residues such as PHE295 and TYR341, which are located within the acyl pocket and the peripheral anionic site. These sites are essential for regulating the entry of acetylcholine into the catalytic gorge, and their predicted interaction with TiO_2_ suggests a potential influence on natural enzymatic turnover.^52–55^

The 100 ns MD-simulation further explains this mechanism. In its native state, AChE maintains a dynamic, flexible structure (RMSD ∼0.20 nm) necessary for its high catalytic rate. However, the predicted interaction with TiO_2_ was associated with a “stabilization” effect, reducing fluctuations to ∼0.15 nm and decreasing the Radius of Gyration. This reduction in conformational flexibility may represent a structural “locking” of the enzyme. While increased stability might seem beneficial for an enzyme, excessive rigidity often alters substrate-binding kinetics. Taken together, the combined docking and simulation results provide a coherent picture in which TiO_2_ interaction stabilizes AChE by restricting its conformational flexibility. Mechanistically, the simulations suggest that this stabilization may arise from sustained interactions within the active site that limit local motions and dampen backbone fluctuations. Consequently, the protein may adopt a more rigid and structurally cohesive conformation, which may have direct implications for its catalytic activity. Thus, results suggest that TiO_2_ may modulate the structural and functional behaviour of AChE. Overall, these findings provide complementary and plausible mechanistic insights into the observed neurotoxicity.

Fig. 8A and B show the cluster heatmap of the biochemical parameters under visible-light and UV-A treatment, respectively. The cluster heatmap revealed that oxidative stress parameters, such as ROS and MDA levels, were closely related to each other under both visible and UV-A treatment. Furthermore, these oxidative stress parameters were closely related to neurotransmitter (AChE) activity under both light treatments. Notably, the analysis revealed that the oxidative stress parameters and neurotransmitter activity were clustered with the survival of *A. salina.*Fig. 8C and D show the Pearson correlation results of the biochemical parameters under visible-light and UV-A illumination, respectively. The Pearson correlation results revealed that survival of the organism was negatively correlated with ROS and MDA content in organisms exposed to either visible-light or UV-A illumination. Similarly, AChE activity displayed a negative correlation with survival under both light treatments. Furthermore, under both light treatments, the ROS generated positively correlated with MDA levels in *A. salina*. Likewise, ROS and MDA production were positively correlated with the AChE activity of the organism under both light treatments. All these results revealed that ROS generation is one of the pivotal modes of action for the detrimental effects in *A. salina*, irrespective of the light treatments. This enhanced ROS generation triggered lipid peroxidation, which was evident from MDA production. These heightened oxidative stress levels may be associated with altered neurotransmitter activity in *A. salina*. Ultimately, oxidative damage and alterations in AChE activity may affect the survival of *A. salina*.

**Fig. 8:**
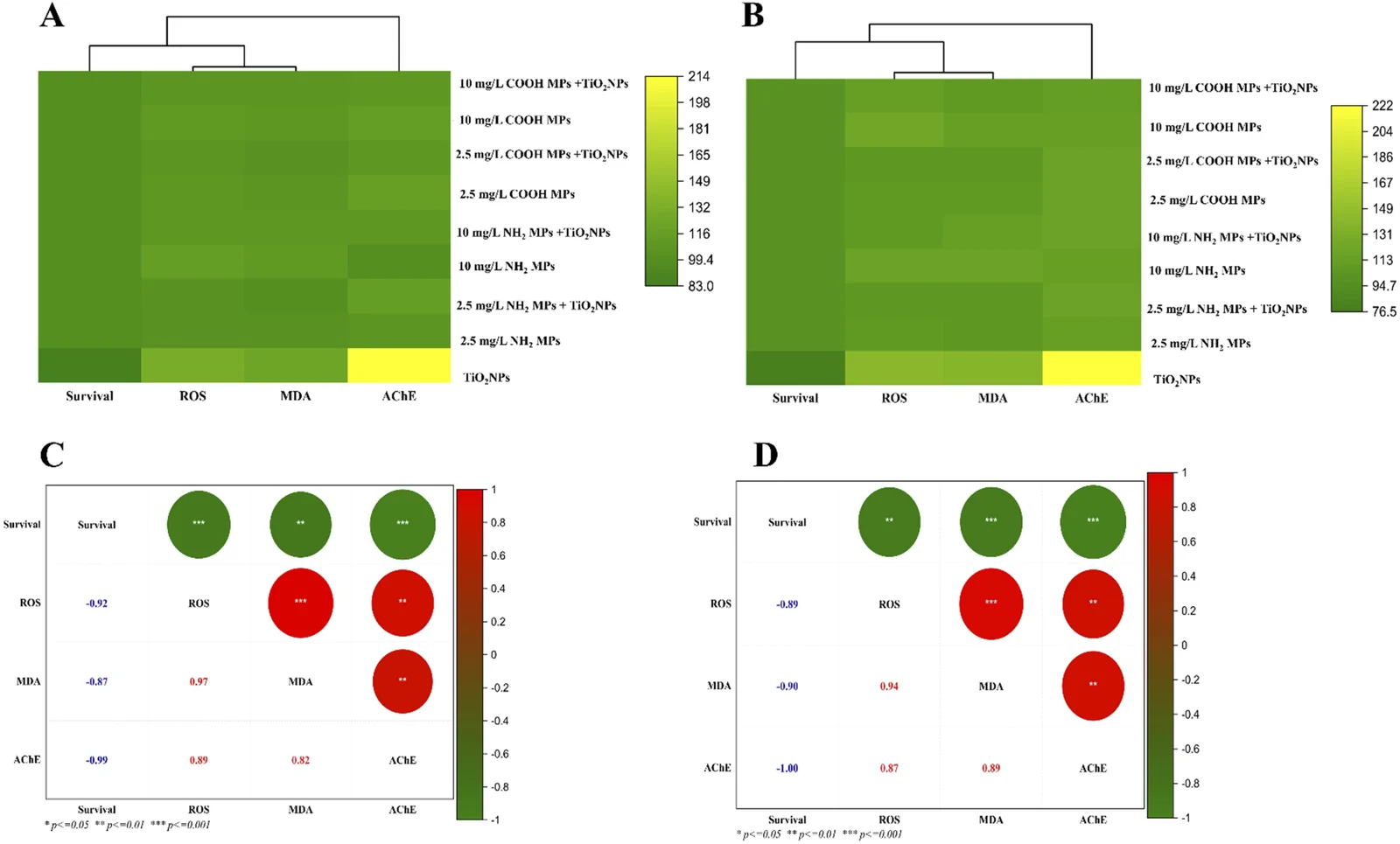
(A) Heatmap analysis of the biological endpoints under visible-light conditions; (B) heatmap analysis of the biological endpoints under UV-A irradiation; (C) Pearson correlation of the biological endpoints under visible-light conditions; (D) Pearson correlation of the biological endpoints under UV-A irradiation.

The depuration experiment revealed lower bioaccumulated Ti levels compared to dietary Ti uptake. This could be attributed to the ability of the organism to eliminate Ti, and it may influence the biomagnification potential. BMF is a quantitative index to evaluate the degree of transfer of a contaminant from a prey to a predator in a food chain.^56^ Notably, both TiO_2_-NPs alone and their co-exposure with PS-MPs exhibited lower BMF (<1), indicating no biomagnification for TiO_2_-NPs alone or TiO_2_-NPs + PS-MPs from *Chlorella* sp. to *A. salina* under the present experimental conditions. This could be attributed to the lower concentration of TiO_2_-NPs used in this study.

Table 2 shows the estimated RQ of TiO_2_-NPs in the marine crustacean *A. salina*. The calculated RQ is a screening-level estimate and is subject to uncertainty associated with extrapolating laboratory-derived toxicity data to environmental conditions using literature-reported environmental concentrations. RQ analysis provides a critical evaluation of the ecological risk posed by the contaminants towards aquatic organisms in the natural environment.^57^ The contaminants exhibiting an RQ value <0.1 are regarded as low ecological risk, <1 is moderate ecological risk, and ≥1 is regarded as high ecological risk. Our findings from the RQ analysis revealed that TiO_2_-NPs pose a high ecological threat to *A. salina* under environmentally relevant concentrations in the marine environment.

**Table 2:** Estimated risk quotient of TiO_2_-NPs in marine crustacean *Artemia salina*

Sl. No	Area	MEC (µg L^−1^)	RQ	References
1	Laizhou Bay	200	34.5	58
2	Jiaozhou Bay	308	53.1	58
3	Mediterranean shores	50	8.6	59
4	Coastal waters of Mallorca	37.6	6.5	60
5	French Mediterranean coast	20 (lower level)	3.4	61
		900 (higher level)	155.1	
6	Mediterranean coast	100 (lower level)	17.2	58
		900 (higher level)	155.1	
7	Jiaozhou Bay sediments	0.697 (lower level)	0.12	62
		2.44 (higher level)	0.42	
8	Mediterranean waters near Marseille	20 (lower level)	3.44	60
		900 (higher level)	155.1	
9	Coastal waters in Majorca beaches	12.1 (lower level)	2.06	63
		37.6 (higher level)	6.48	

## 5. Conclusion

The findings of this study revealed that exposure to TiO_2_-NPs alone resulted in the highest Ti uptake in microalgae *Chlorella* sp. compared to TiO_2_-NPs + PS-MPs. Furthermore, the bio-uptake of Ti was more in *Chlorella* sp. under UV-A than under visible-light treatments. These trends were well aligned with Ti bio-uptake in *A. salina* following dietary exposure to contaminated microalgae. *A. salina* fed with microalgae pre-treated with TiO_2_-NPs alone showed a significant decline in survival, with a more pronounced decline under UV-A treatments. The microalgae exposed to TiO_2_-NPs alone and in combination with PS-MPs, showed elevated ROS and MDA levels in *A. salina*, with a more pronounced elevation observed in the TiO_2_-NPs alone dietary exposed group. This enhanced oxidative stress may be associated with impaired AChE activity, and the concurrent impact of these two parameters may adversely impact the survival of dietary-exposed *A. salina*. Under both light treatments, the BMF was less (<1) for TiO_2_-NPs in both the presence and absence of PS-MPs, indicating reduced risk of biomagnification. RQ analysis revealed a high risk potential of TiO_2_-NPs in the marine system. Overall, this study elucidates the significance of light treatments and the presence of co-contaminants (PS-MPs) in modulating the trophic transfer potential of TiO_2_-NPs from microalgae to brine shrimp. Future studies should investigate how environmental factors such as ocean acidification, ocean warming, and natural organic matter influence contaminant behavior and toxicity. Furthermore, future studies can extend this investigation to higher trophic level organisms like fish to better understand their potential risk in the marine food chain.

## Author contributions

Camil Rex M: investigation, methodology, visualization, conceptualization, formal analysis, and writing – original draft; Manshi Kumari Gupta: methodology and writing – original draft; Chinnappan Sudandiradoss: methodology, validation, formal analysis, and writing – review and editing; Amitava Mukherjee: conceptualization, methodology, supervision, validation, formal analysis, project administration, writing – review and editing.

## Conflicts of interest

There are no conflicts to declare.

## Supplementary Material

RA-016-D6RA05133B-s001

## Data Availability

The data supporting this article have been included as part of the supplementary information (SI). Supplementary information is available. See DOI: https://doi.org/10.1039/d6ra05133b.
