# Supplementary material for: Polystyrene microplastics reduce the food chain transfer and toxicity of TiO2 nanoparticles in crustacean Artemia salina through dietary exposure of microalga Chlorella sp.: impact of visible light and ultraviolet-A radiation
Source: RSC Adv. 2026 Aug 18;16(45):56885–98. doi: 10.1039/d6ra05133b (PMC13484639; doi:10.1039/d6ra05133b)
Supplement: Supplementary file 1 [file RA-016-D6RA05133B-s001.pdf]

Supplementary Information (SI) for RSC Advances.
This journal is © The Royal Society of Chemistry 2026

Polystyrene Microplastics Reduce the Food Chain Transfer and Toxicity of TiO_2 Nanoparticles in Crustacean *Artemia salina* through Dietary Exposure of Microalga *Chlorella* sp.: Impact of Visible Light and Ultraviolet-A Radiation

Camil Rex M [a], Manshi Kumari Gupta [b], Chinnappan Sudandiradoss [b], and Amitava Mukherjee [a*]

[a] *Centre for Nanobiotechnology, VIT, Vellore, Tamil Nadu, India*

[b] *Department of Biotechnology, School of Bio Sciences and Technology, VIT, Vellore, Tamil Nadu, India*

*** Corresponding Author**

Amitava Mukherjee

Senior Professor

Centre for Nanobiotechnology

Vellore Institute of Technology, Vellore 632014, India

Email: amitav@vit.ac.in, amit.mookerjea@gmail.com

Table S1. Composition of Artificial Sea Water per 1000 ml

Chemicals	Grams
Sodium Chloride	26.29
Potassium chloride	0.74
Calcium chloride	0.99
Magnesium chloride	6.09
Magnesium sulphate heptahydrate	3.94

Method S1: Chemicals used

Cell-permeable non-fluorescent probe 2′, 7′ dichlorofluorescein diacetate (DCFH-DA) was supplied by Sigma-Aldrich, USA. Other chemicals, such as 5, 5′-dithiobis (2-nitrobenzoic acid), trichloroacetic acid (TCA), acetylthiocholine iodide, ethylenediaminetetraacetic acid (EDTA), and thiobarbituric acid (TBA) were supplied by Hi-Media Pvt. Ltd., India. Nitroblue tetrazolium chloride (NBT) and hydrogen peroxide solution (H2O2- 30% w/v) were purchased from SDFCL, Mumbai, India. Triton X-100 and sodium carbonate (Na_2CO3) were purchased from Sigma Aldrich.

Method S2: Oxidative Stress estimation in dietary-exposed *Artemia salina.*

The generation of ROS in control and test samples of *Artemia salina* following dietary exposure to TiO_2, PSMPs, and their binary mixture was performed using 2',7'-dichlorodihydrofluorescein diacetate as detailed by [1]. Specifically, 80 µL of the homogenized samples were incubated with 20 µL of DCFHDA for 30 minutes in the dark. The fluorescence intensity was measured using a fluorescent spectrophotometer (Cary Eclipse, Agilent Technologies, USA) with an excitation wavelength of 485 nm and an emission wavelength of 530 nm.

Malondialdehyde (MDA) levels in *Artemia salina* following dietary exposure to TiO_2, PSMPs, and their binary mixture were assessed using a TCA–TBA assay. TCA–TBA mixture (2 ml) was added to the homogenized samples and vortexed before being heated at 90°C for 30 minutes. After cooling, the samples were centrifuged at 7,000 rpm, and absorbance was measured at 532 nm and 600 nm using a microplate reader [2].

Method S3: In vitro AChE activity

The cholinesterase enzyme activity in *Artemia salina* following dietary exposure to TiO_2, PSMPs, and their binary mixture was estimated using the protocol described by [3]. Briefly, the nauplii from control and test groups were homogenized and centrifuged at 7,000 rpm for 10 minutes at 4°C. Furthermore, 50 μL of the supernatant was mixed with 10 μL of acetylthiocholine iodide (10 mM), 10 μL of 5,5′-dithiobis (2-nitrobenzoic acid) (5 mM), and 30 μL of potassium phosphate buffer (100 mM, pH 7.4). A blank control was prepared by replacing 5,5′-dithiobis (2-nitrobenzoic acid) with potassium phosphate buffer. Absorbance was recorded at 405 nm every minute for 3 minutes using a microplate reader [4].

Method S4: *In silico* molecular docking and molecular dynamics simulation of TiO_2 with AChE

The geometry of the ligand was subsequently optimized via Avogadro [5], employing the MMFF94s force field to achieve a minimized energy conformation suitable for docking simulations. Prediction of ligand-accessible binding pockets on protein structures was carried out using PrankWeb [6], which leverages convolutional neural networks trained on known protein-ligand complexes, and CASTp 3.0 [6], which applies alpha shape theory to identify and quantify topographic surface features, such as pocket volume and area. Molecular docking was conducted using GNINA version 1.3 [7], an advanced docking tool that integrates traditional AutoDock Vina scoring with deep learning–based convolutional neural network (CNN) models, providing enhanced accuracy in ligand pose ranking and binding affinity estimation, to identify potential interaction sites between AChE and the representative atomistic TiO_2 model. Following docking, LigPlot$^+$ [8] was employed to visualize protein-ligand interaction profiles, enabling the identification of critical hydrogen bonding, hydrophobic interactions, and other non-covalent contacts that define the structural basis of TiO_2 binding. This integrated computational workflow provides preliminary structural insights into potential TiO_2–protein interactions at the atomic level and serves as an exploratory framework for identifying potential interaction sites.

Probing the Conformational Dynamics of AChE-TiO2 Complex via GROMACS-Based Molecular Dynamics Simulation

To interrogate the structural dynamics and conformational resilience of TiO_2-protein complex involving AChE protein, all-atom molecular dynamics (MD) simulations were executed using GROMACS 2024.3 (https://www.gromacs.org/) [9]. Protein topologies were generated employing the CHARMM36m force field, which provides enhanced backbone sampling

fidelity, while titanium dioxide (TiO_2) nanoparticle parameters were integrated through Lennard-Jones (LJ) potentials based on prior empirical derivations [10]. The non-bonded interaction potential for TiO_2 atoms was modeled using the Lennard-Jones 12-6 potential, where σ denotes the finite interatomic distance at which the inter-particle potential is zero (reflecting the effective atomic radius), and ε characterizes the well depth, representing the strength of van der Waals attraction. These parameters are highly sensitive to the local coordination environment and electronic density, necessitating accurate tabulation. Specifically, σ-values have been well established for lighter elements, and herein the LJ parameters were adopted from Luan et al. (2015), optimized for inorganic–biological hybrid interfaces. The parametric values employed were: ε (Ti^{4+}) = 1.100 kJ/mol, σ (Ti^{4+}) = 0.196 nm, and ε (O^{2-}) = 0.902 kJ/mol, σ (O^{2-}) = 0.292 nm [10, 11]. System solvation was performed using the TIP3P explicit water model, and counterions (Na^+ and Cl^-) were introduced to neutralize net charge and emulate physiological ionic strength. The protein-nanoparticle complexes were centered in a triclinic box with a 1.0 nm solvent padding, and energy minimization was achieved via the steepest descent algorithm for 50,000 steps, converging at a force tolerance of 1000 kJ/mol/nm. During both minimization and equilibration, position restraints were enforced on the TiO_2 core to prevent artificial structural drift. The system underwent dual-phase equilibration, beginning with a 100 ps NVT ensemble at 300 K using the modified Berendsen thermostat (V-rescale) for temperature coupling, followed by a 100 ps NPT ensemble at 1 atm pressure, controlled via the Parrinello-Rahman barostat, facilitating pressure relaxation. Long-range electrostatics were resolved using the Particle Mesh Ewald (PME) algorithm, with both electrostatic and van der Waals cutoffs set to 1.0 nm. Bond constraints for all hydrogen-containing interactions were applied via the LINCS algorithm, enabling the use of a 2 fs integration timestep. The production MD simulation was run for 100 nanoseconds (ns) with a 2 femtosecond (fs) integration time step, and trajectory frames were recorded every 100 ps.

Structural and thermodynamic analyses included the calculation of root mean square deviation (RMSD), root mean square fluctuation (RMSF), and radius of gyration (Rg) to monitor conformational stability, residue flexibility, and compactness, respectively. The trajectory visualizations were handled using Visual Molecular Dynamics (VMD) (https://www.ks.uiuc.edu/Research/vmd/) [12], graph plotting was performed in Xmgrace (https://plasma-gate.weizmann.ac.il/Grace/) [13], and the final structural models were visualized using PyMOL [14].

Method S5: Risk quotient

Risk quotient is an approach to determine the risk posed by the contaminants in the aquatic system. In this study, the ecological risk posed by the TiO_2 NPs was estimated using risk quotient analysis. To determine the risk quotient of TiO_2 NPs, the following formula was used,

Predicted no-effect concentration = EC_{50} / Assessment factor (1)

RQ = Measured environmental concentration / Predicted no-effect concentration (2)

In the current work, the measured environmental concentrations (MEC) were taken from the published literature (Table 2). The EC_{50} value (5800 µg/L) of TiO_2 NPs on exposure to Artemia salina was calculated. The predicted no-effect concentration (PNEC) was calculated based on the EC_{50} values by applying an assessment factor (AF). The PNEC was determined to be 5.8 µg/L. The AF was set at 1000 in the present work, as per the European Chemical Agency, while conducting the acute toxicity [15].

Figures

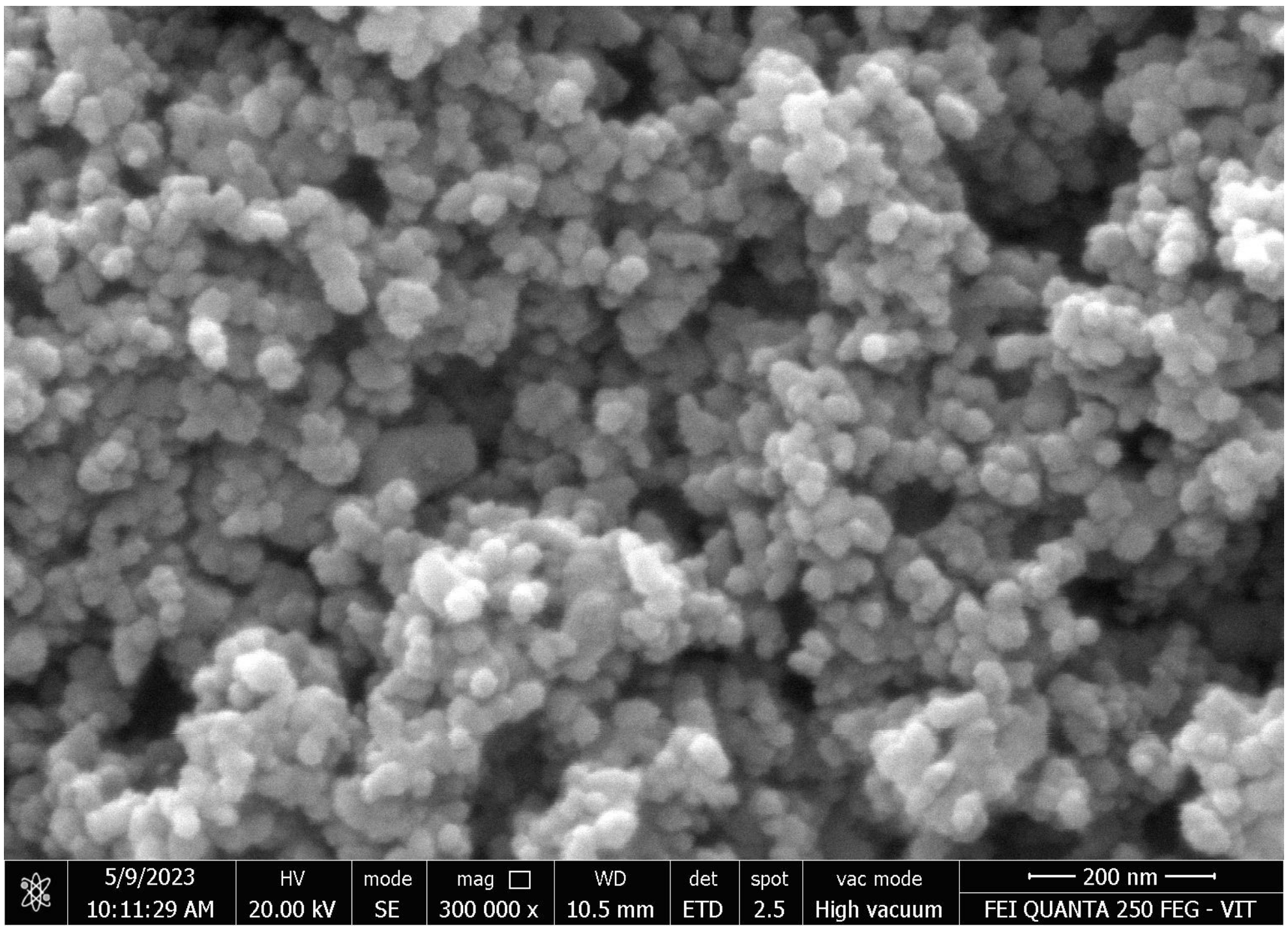

Fig. S1 FE-SEM image of TiO_2 NPs

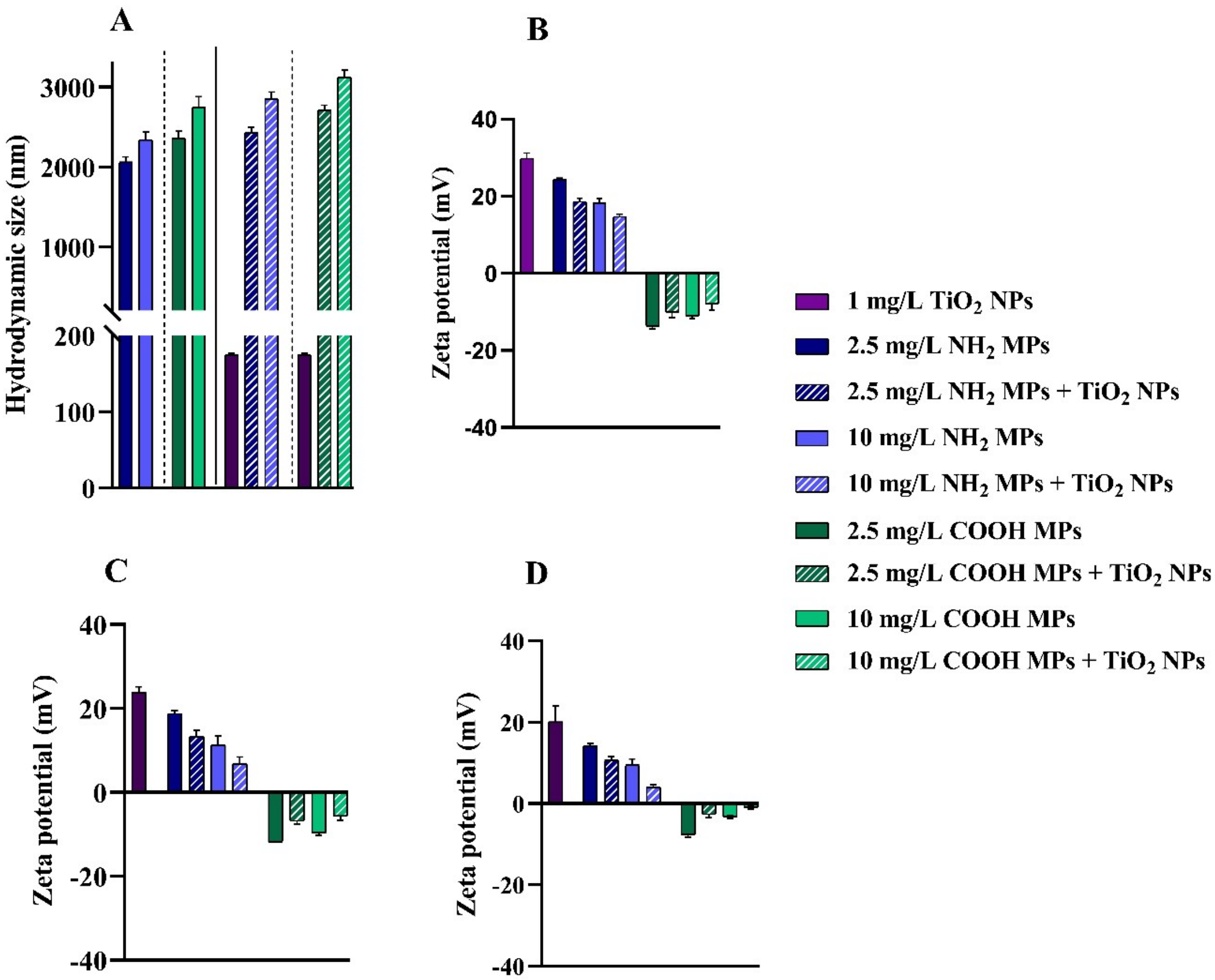

Fig. S2. (A) Initial hydrodynamic size and (B) initial zeta potential of TiO_2 NPs, PS-MPs, and their co-exposure groups in ASW; final (48 h) zeta potential of TiO_2 NPs, PS-MPs, and their co-exposure groups in ASW under (C) visible-light and (D) UV-A conditions.

References

1. P. A. Athulya, Z. Sunil, S. Manzo and N. Chandrasekaran, Prepared microplastics interaction with Artemia salina under low pH conditions representing ocean acidification; a simulated environmental exposure, *Journal of Environmental Management*, 2023, **348**, 119367.
2. C. Rex M, A. Bairoliya, S. Gairola, P. Sureshkumar, V. Niranjan, A. Nag and A. Mukherjee, Engineered biocorona on microplastics as a toxicity mitigation strategy in marine environment: Experiments with a marine crustacean Artemia salina, *Journal of Hazardous Materials Advances*, 2025, **17**, 100558.
3. F. Cavion, L. Fusco, S. Sosa, C. Manfrin, B. Alonso, A. Zurutuza, R. Della Loggia, A. Tubaro, M. Prato and M. Pelin, Ecotoxicological impact of graphene oxide: Toxic effects on the model organism Artemia franciscana, *Environmental Science: Nano*, 2020, **7**, 3605-3615.
4. V. Thiagarajan, R. Seenivasan, D. Jenkins, N. Chandrasekaran and A. Mukherjee, Mixture toxicity of TiO2 NPs and tetracycline at two trophic levels in the marine ecosystem: Chlorella sp. and Artemia salina, *Science of The Total Environment*, 2022, **812**, 152241.
5. M. D. Hanwell, D. E. Curtis, D. C. Lonie, T. Vandermeersch, E. Zurek and G. R. Hutchison, Avogadro: an advanced semantic chemical editor, visualization, and analysis platform, *Journal of cheminformatics*, 2012, **4**, 17.
6. L. Jendele, R. Krivak, P. Skoda, M. Novotny and D. Hoksza, PrankWeb: a web server for ligand binding site prediction and visualization, *Nucleic acids research*, 2019, **47**, W345-W349.
7. A. T. McNutt, Y. Li, R. Meli, R. Aggarwal and D. R. Koes, GNINA 1.3: the next increment in molecular docking with deep learning, *Journal of Cheminformatics*, 2025, **17**, 28.
8. R. A. Laskowski and M. B. Swindells, LigPlot+: multiple ligand–protein interaction diagrams for drug discovery.*Journal*, 2011.
9. S. Pronk, S. Páll, R. Schulz, P. Larsson, P. Bjelkmar, R. Apostolov, M. R. Shirts, J. C. Smith, P. M. Kasson and D. Van Der Spoel, GROMACS 4.5: a high-throughput and highly parallel open source molecular simulation toolkit, *Bioinformatics*, 2013, **29**, 845-854.
10. B. Luan, T. Huynh and R. Zhou, Simplified TiO2 force fields for studies of its interaction with biomolecules, *The Journal of chemical physics*, 2015, **142**.
11. E. G. Brandt and A. P. Lyubartsev, Systematic optimization of a force field for classical simulations of TiO2–water interfaces, *The Journal of Physical Chemistry C*, 2015, **119**, 18110-18125.
12. W. Humphrey, A. Dalke and K. Schulten, VMD: visual molecular dynamics, *Journal of molecular graphics*, 1996, **14**, 33-38.
13. M. J. Abraham, T. Murtola, R. Schulz, S. Páll, J. C. Smith, B. Hess and E. Lindahl, GROMACS: High performance molecular simulations through multi-level parallelism from laptops to supercomputers, *SoftwareX*, 2015, **1**, 19-25.

14. R. E. Rigsby and A. B. Parker, Using the P y MOL application to reinforce visual understanding of protein structure, *Biochemistry and Molecular Biology Education*, 2016, **44**, 433-437.
15. A. Debroy, M. J. Nirmala, M. Pulimi, W. J. Peijnenburg and A. Mukherjee, Assessing the role of the graphene family nanomaterials (GFNs: Graphene, GO, rGO) in modifying the toxicity potential and environmental risk of flame retardant, tetrabromobisphenol-A (TBBPA) in the marine microalgae Chlorella sp, *Chemosphere*, 2024, **361**, 142491.
